# Microscopy-BIDS: An Extension to the Brain Imaging Data Structure for Microscopy Data

**DOI:** 10.3389/fnins.2022.871228

**Published:** 2022-04-19

**Authors:** Marie-Hélène Bourget, Lee Kamentsky, Satrajit S. Ghosh, Giacomo Mazzamuto, Alberto Lazari, Christopher J. Markiewicz, Robert Oostenveld, Guiomar Niso, Yaroslav O. Halchenko, Ilona Lipp, Sylvain Takerkart, Paule-Joanne Toussaint, Ali R. Khan, Gustav Nilsonne, Filippo Maria Castelli, The BIDS Maintainers, Stefan Appelhoff, Julien Cohen-Adad

**Affiliations:** ^1^NeuroPoly Lab, Institute of Biomedical Engineering, Polytechnique Montreal, Montreal, QC, Canada; ^2^Kwanghun Chung Lab, Picower Institute for Learning and Memory, Massachusetts Institute of Technology, Cambridge, MA, United States; ^3^McGovern Institute for Brain Research, Massachusetts Institute of Technology, Cambridge, MA, United States; ^4^Department of Otolaryngology–Head and Neck Surgery, Harvard Medical School, Boston, MA, United States; ^5^National Research Council, National Institute of Optics, Sesto Fiorentino, Italy; ^6^European Laboratory for Non-Linear Spectroscopy (LENS), Sesto Fiorentino, Italy; ^7^Nuffield Department of Clinical Neurosciences, Wellcome Centre for Integrative Neuroimaging, FMRIB, University of Oxford, Oxford, United Kingdom; ^8^Department of Psychology, Stanford University, Stanford, CA, United States; ^9^Donders Institute for Brain, Cognition and Behaviour, Radboud University, Nijmegen, Netherlands; ^10^NatMEG, Department of Clinical Neuroscience, Karolinska Institutet, Stockholm, Sweden; ^11^Psychological and Brain Sciences, Indiana University, Bloomington, IN, United States; ^12^Department of Psychological and Brain Sciences, Center for Open Neuroscience, Dartmouth College, Hanover, NH, United States; ^13^Department of Neurophysics, Max Planck Institute for Human Cognitive and Brain Sciences, Leipzig, Germany; ^14^Institut de Neurosciences de la Timone, CNRS–Aix Marseille Université, Marseille, France; ^15^Department of Neurology and Neurosurgery, Faculty of Medicine and Health Sciences, Montreal Neurological Institute and Hospital, McGill University, Montreal, QC, Canada; ^16^Robarts Research Institute, University of Western Ontario, London, ON, Canada; ^17^Department of Clinical Neuroscience, Karolinska Institutet, Stockholm, Sweden; ^18^Swedish National Data Service, Gothenburg University, Gothenburg, Sweden; ^19^Mila – Quebec AI Institute, Montreal, QC, Canada; ^20^Functional Neuroimaging Unit, Centre de Recherche de l’Institut Universitaire de Montréal (CRIUGM), Université de Montréal, Montreal, QC, Canada

**Keywords:** microscopy, open science, data structure, data sharing, specification, standardization

## Abstract

The Brain Imaging Data Structure (BIDS) is a specification for organizing, sharing, and archiving neuroimaging data and metadata in a reusable way. First developed for magnetic resonance imaging (MRI) datasets, the community-led specification evolved rapidly to include other modalities such as magnetoencephalography, positron emission tomography, and quantitative MRI (qMRI). In this work, we present an extension to BIDS for microscopy imaging data, along with example datasets. Microscopy-BIDS supports common imaging methods, including 2D/3D, *ex*/*in vivo*, micro-CT, and optical and electron microscopy. Microscopy-BIDS also includes comprehensible metadata definitions for hardware, image acquisition, and sample properties. This extension will facilitate future harmonization efforts in the context of multi-modal, multi-scale imaging such as the characterization of tissue microstructure with qMRI.

## Introduction

Microscopy is widely used in neuroscience to characterize tissue microstructure and study neurological pathologies. Given the variety of imaging technologies (e.g., optical, electron, and micro-CT) and variability in data formats, organizing and sharing microscopy datasets poses multiple challenges. In addition to imaging data, well-documented information about the experimental protocol for image acquisition and sample preparation, the hardware specifications, and the processing pipelines are needed to ensure transparency when sharing data objects and promote the reusability of the datasets (Hammer et al., 2021; Huisman et al., 2021; Ropelewski et al., 2021). A predictable, consistent and intuitive data structure (i.e., folder hierarchy, file naming) is also desirable to facilitate both interactive and automated processes for data curation, indexing, and processing, and contributes to the FAIR principles (Wilkinson et al., 2016) of *interoperability* and *reusability* (Bandrowski et al., 2021; Huisman et al., 2021). Collecting and curating increasingly large and complex microscopy datasets is time- and resource-consuming, highlighting the need for a sharing standard. As the information required differs depending on the modalities and applications, an ideal standard should find a balance between usability/simplicity and completeness/complexity to promote adoption while remaining flexible enough to evolve with technological advancements and future community needs (Hammer et al., 2021; Huisman et al., 2021; Sarkans et al., 2021).

The microscopy community has yet to settle on a universal standard specification for metadata reporting (Hammer et al., 2021; Huisman et al., 2021), but several groups and initiatives are working on standardization for data structure, metadata, and/or quality control to tackle these challenges. Those efforts include the Stimulating Peripheral Activity to Relieve Conditions (SPARC) Dataset Format (Bandrowski et al., 2021), A perspective on Microscopy Metadata: data provenance and quality control (Huisman et al., 2021), the Essential Metadata for 3D BRAIN Microscopy (Ropelewski et al., 2021) with metadata standards for 3D microscopy datasets for use by the Brain Research through Advancing Innovative Neurotechnologies (BRAIN) Initiative^1^ and implemented in the Brain Image Library (BIL) (Benninger et al., 2020), the Recommended Metadata for Biological Images (REMBI) (Sarkans et al., 2021) for light and electron microscopy, the open Metadata Initiative for Neuroscience Data Structures (openMINDS)^2^ and the Quality Assessment and Reproducibility for Instruments & Images in Light Microscopy (QUAREP-LiMi) (Boehm et al., 2021). Recent developments also include the 4DN-BINA-OME framework (Hammer et al., 2021), a tier-based microscopy metadata specification extending the Open Microscopy Environment (OME) Data Model (Goldberg et al., 2005)^3^ by the 4D Nucleome Initiative (4DN) (Dekker et al., 2017)^4^ in collaboration with BioImaging North America (BINA),^5^ along with metadata collection tools (Kunis et al., 2021; Rigano et al., 2021; Ryan et al., 2021). The OME community has also been developing the Next Generation File Format (NGFF), which uses a Zarr-based format for dealing more flexibly with the different scales of microscopy data (Moore et al., 2021).

In the neuroimaging field, the Brain Imaging Data Structure (BIDS) (Gorgolewski et al., 2016)^6^ is now a well-established specification for organizing and sharing data. First developed for magnetic resonance imaging (MRI) datasets, BIDS was extended to several other imaging modalities and applications such as magnetoencephalography (Niso et al., 2018), positron emission tomography (Knudsen et al., 2020; Norgaard et al., 2022), and quantitative MRI (qMRI) (Karakuzu et al., 2021). The popularity of BIDS and its wide adoption stems from the fact that it is both a data structure and it defines human- and machine-readable metadata, which facilitates the interpretation and sharing of imaging datasets. The open-source community revolving around the BIDS standard is also actively contributing to conversion and dataset validation tools, as well as automated analysis pipelines^7,^^8,^^9^.

In this work, we present Microscopy-BIDS, an extension to BIDS for microscopy data for several 2D and 3D imaging scenarios, and discuss its compatibility with other initiatives. Microscopy-BIDS defines a standardized structure and naming scheme for raw microscopy data and aims to cover the most common use cases. It also incorporates key metadata for hardware, image acquisition, and sample properties. Furthermore, as Microscopy-BIDS follows the BIDS common principles, the standardized data structure across imaging modalities will facilitate the implementation of multi-modal imaging analysis, for example performing histological validation of qMRI metrics or developing multiscale registration algorithms. The full Microscopy-BIDS specification is available on the BIDS website^10^.

## Microscopy-BIDS Specification

### Overview

**FIGURE 1:**
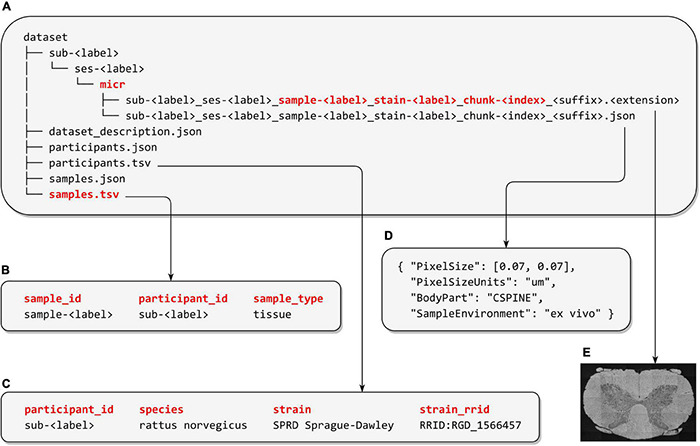
Microscopy-BIDS overview. **(A)** Microscopy-BIDS dataset hierarchy. The raw imaging data files and their sidecar JSON files are stored in the “sub- < *label* >” directory, followed by the optional “ses- < *label* >” (session) directory and then the data type “micr” directory. The new “sample- < *label* >”, “chunk- < *label* >”, and “stain- < *label* >” entities are shown in red in the image filename. **(B)** Example of “samples.tsv” file describing sample attributes such as “sample_type”. **(C)** Example of “participants.tsv” file including animal metadata (“species”, “strain”, and “strain_rrid”). **(D)** Example of sidecar JSON metadata file. **(E)** Raw microscopy imaging data modified from Zaimi et al. (2018) under Creative Commons Attribution 4.0 International License (http://creativecommons.org/licenses/by/4.0/).

The Microscopy-BIDS data structure follows the established BIDS hierarchy and naming conventions. The extension required the addition of new filename entities to properly detail specific properties of microscopy data: “sample,” “stain,” and “chunk” described in the next sections. In each subject’s folder, the raw microscopy data are placed under the optional session directory, then in the “micr” data type directory, accompanied by a sidecar JSON file with additional metadata (see Figure 1). At the root of the dataset, together with the “dataset_description.json,” and the recommended “participants.tsv” and “participants.json” files, a new “samples.tsv” file, with its corresponding sidecar JSON file, have been added to describe samples attributes (e.g., “sample_type”). Three new columns are also added and recommended in the existing “participants.tsv” file to describe animal metadata (“species,” “strain,” and “strain_rrid”).

The filename <suffix> represents the specific microscopy imaging modality. The included microscopy modalities in Microscopy-BIDS and their suffixes are: transmission electron microscopy (TEM), scanning electron microscopy (SEM), Micro-CT (uCT), bright-field microscopy (BF), dark-field microscopy (DF), phase-contrast microscopy (PC), differential interference contrast microscopy (DIC), fluorescence microscopy (FLUO), confocal microscopy (CONF), polarized-light microscopy (PLI), coherent anti-Stokes Raman spectroscopy (CARS), two-photon excitation microscopy (2PE), multi-photon excitation microscopy (MPE), super-resolution microscopy (SR), non-linear optical microscopy (NLO), optical coherence tomography (OCT), and selective plane illumination microscopy (SPIM).

Microscopy imaging data can be stored in various file formats. Constraining BIDS datasets to support a few selected raw data formats facilitates the handling of datasets and the future development of software applications to process them. Suitable and popular file formats for storing the raw microscopy data were selected to satisfy three main considerations: (i) accommodate datasets stored in 2D image formats and whole-slide imaging formats, (ii) accommodate lossless and lossy compression, and (iii) avoid unnecessary conversions of the original data between tiled and non-tiled format.

To meet the above criteria, Microscopy-BIDS supports three file formats. For non-tiled 2D data, the PNG and TIFF were chosen (<*extension*> “.png” and “.tif,” respectively). They are both conveniently readable by various image editing toolboxes. PNG files offer lossless compression with a smaller file size compared to uncompressed TIFF, whereas TIFF files allow for lossy compression as well. For large resolution whole-slide imaging and 3D data, the standardized file structure OME-TIFF developed by the OME consortium was chosen (<*extension*> “.ome.tif” and “.ome.btf” for regular TIFF and BigTIFF files, respectively). OME-TIFF allows for storage of tiled and multi-dimensional 5D+ data.^11^^,^ ^12^ It includes metadata from the OME Data Model (Goldberg et al., 2005)^13^ in an OME-XML header, and files from different proprietary formats can be interpreted and converted to OME-TIFF *via* the *Bio-Formats*^14^ library. Other specialized toolboxes to process OME-TIFF include *tifffile*,^15^ *libvips*,^16^ *apeer-ometiff-library*,^17^ and *pyometiff.*^18^ As OME-TIFF contains higher-dimensional information, the Microscopy-BIDS specification could include higher dimensions (e.g., time) as their usage becomes more common.

### The “Sample” Entity and the “samples.tsv” File

Microscopy-BIDS follows the common principles and definitions of BIDS such as *subject*, *session*, *data acquisition*, and *run*. BIDS defines the subject as “a person or animal participating in the study”^19^ (e.g., a human, a mouse). In the context of microscopy, the primary entity studied is often a sample extracted from a subject and not the subject itself. However, to ensure compatibility with other BIDS modalities, a subject retains the same definition as in BIDS. Therefore, to describe several samples pertaining to the same subject, we introduced the “sample” entity to the specification.

The sample attributes are described in the new “samples.tsv” file. It includes a mandatory “sample_type” column which specifies the type of sample such as “tissue,” “primary cell,” or “cell-free sample,” from *ENCODE Biosample Type.*^20^ Two other sample attributes are recommended: (i) “pathology” and (ii) “derived_from” that indicates when a sample is derived from another sample (e.g., a slice extracted from a block of tissue). The “samples.tsv” file is reserved for sample attributes whereas subject attributes such as age and sex are described in “participants.tsv” as per BIDS common principles.

### The “Chunk” Entity

**FIGURE 2:**
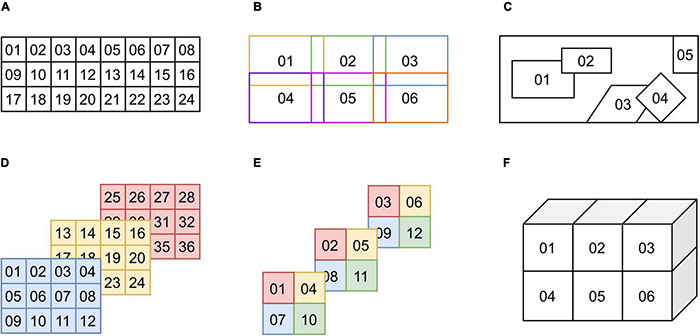
Examples of chunk configurations. **(A)** Ordered 2D chunks without overlap. **(B)** Ordered 2D chunks with overlap. **(C)** Unordered 2D chunks with and without overlap. **(D,E)** Ordered 2D chunks on different 3D planes. **(F)** Ordered 3D chunks.

In addition to describing multiple samples per subject, Microscopy-BIDS introduces the “chunk” entity to describe different regions imaged from the same physical sample under the microscope in the same imaging experiment. In the context of microscopy, a single sample can be acquired through a series of images or volumes with different fields of view. As such, the “chunk-<index>” is used in the filename to distinguish between these different chunks. Examples of different ordered and unordered chunk configurations with and without overlaps are presented in Figure 2. In the case of ordered chunks, it is recommended to describe the spatial relationship between the chunks, to reconstruct the image or volume, as an affine transformation matrix in the JSON sidecar files (“ChunkTransformationMatrix” and “ChunkTransformationMatrixAxis” fields) reporting scaling and translation along the “X,” “Y,” and “Z” axis.

### The “Stain” Entity

Microscopy-BIDS also includes a new “stain” entity. It is used to distinguish files from the same sample using different stains or antibodies for contrast enhancement (staining and immunostaining). In addition to the “stain” entity, three fields in the JSON sidecar metadata files are recommended to describe the sample staining and/or primary and secondary antibodies used (“SampleStaining,” “SamplePrimaryAntibody,” and “SampleSecondaryAntibody” fields).

### Animal Metadata

To date, the BIDS specification has been focusing mainly on humans. As part of the development of Microscopy-BIDS, and in collaboration with the animal electrophysiology BIDS extension proposal (BEP032), we introduced three new columns recommended for the “participants.tsv” file to include animal metadata. The first one is “species” with the binomial species name from the *NCBI Taxonomy*^21^ (e.g., “mus musculus,” “rattus norvegicus”). We also added the “strain” and “strain_rrid” columns, respectively corresponding to the name of the strain of the species and its research resource identifier (RRID)^22^.

### Microscopy Metadata

Microscopy-BIDS includes required, recommended and optional metadata fields, stored in JSON sidecar files, covering four critical aspects of microscopy image acquisition: (i) Device Hardware, with fields such as “Manufacturer” or “InstitutionName” of the equipment used, (ii) Image Acquisition, with fields such as “PixelSize,” “Magnification,” and “ImageAcquisitionProtocol,” (iii) Sample, with fields such as “BodyPart,” “SampleEnvironment,” “SampleFixation,” and “SampleExtractionProtocol,” and (iv) Chunk Transformations, which describe the spatial location of each “chunk” to reconstruct a full image or volume.

In particular, the “BodyPart” field is used to describe the anatomical location of the sample using controlled vocabulary from the *DICOM Body Part Examined*^23^ and allows for the description of non-brain structures. To cover different imaging methods and scenarios, the “SampleEnvironment” metadata field indicates if the samples were acquired “*ex vivo*,” “*in vivo*,” or “*in vitro*”. Other dedicated metadata fields are also included to specify the sample extraction protocol and sample extraction institution when different from the institution acquiring the images (“SampleExtractionProtocol” and “SampleExtractionInstitution” fields). Furthermore, for image acquisition, an “OtherAcquisitionParameters” field was included allowing the description of relevant image acquisition parameters that are not otherwise present in the specification.

### Validation and Example Datasets

In parallel to the development of Microscopy-BIDS specification, we implemented an extension for microscopy to the *bids-validator*^24^ with two example datasets^25^ on the *bids-examples* repository. These example datasets contain the main features of Microscopy-BIDS such as the PNG and OME-TIFF file formats, the “sample,” “stain,” and “chunk” entities, and various metadata, including animal metadata and the new “chunk transformations” concept. These examples can serve as a guide for the curation of new microscopy datasets compatible with BIDS. In addition to these examples, two “real-world” datasets following Microscopy-BIDS were put together and publicly shared. One is based on scanning electron microscopy (SEM) (Zaimi et al., 2021) and the other is based on selective plane illumination microscopy (SPIM) (Mazzamuto et al., 2021). Additional microscopy datasets related to human brain whole hemisphere and Broca’s area immunostaining and associated MRI data are being released through the DANDI data archive^26,^^27^.

## Discussion

We presented a new extension to BIDS for microscopy data. Fully compatible with the BIDS specification, the extension defines file formats that are suitable for the storage of 2D and 3D microscopy data of popular modalities. Microscopy-BIDS also introduces the new “sample,” “stain,” and “chunk” key concepts to describe microscopy data, as well as comprehensible metadata and new additions to BIDS for animal metadata.

Introducing the “sample” concept was pivotal in the development of the proposal as keeping the BIDS “subject” definition intact is important for consistency between imaging modalities. For example, a “subject” in MRI must represent the same “physical entity” as a “subject” in microscopy. Therefore, the “sample” entity allows distinguishing multiple samples from the same subject, which happens, for example, in the case of a biopsy procedure or serial sectioning. A sample could have been described by other words such as “tissue” or “cell,” but we chose to combine those concepts in the unique term “sample” to avoid adding undue complexity to the data scheme. Additional sample attributes can then be described in the new “samples.tsv” file with the required column “sample_type” and other optional columns as appropriate. To avoid any ambiguity, both the “subject” and “sample” entities are required in the filenames of microscopy data.

Another challenge was that a single microscopy sample may be imaged with multiple individual files, for which we introduced the “chunk” filename entity. The name itself was chosen as best representing a “portion” of a physical sample when acquired through a series of images or volumes (e.g., in z-scanning). We considered many mechanisms to describe the chunk’s spatial coordinates and transformations. Because of the complexity and plurality of use cases, we chose to focus on the image or volume reconstruction from the chunks. The description of this transformation is achieved with an affine matrix in the existing JSON sidecar metadata, in the implicit coordinate system of the transformation itself. Transformations related to processing pipelines are not covered by Microscopy-BIDS and should be described in derivatives. Future developments include the description of coordinate systems for microscopy data and spatial mappings for registration with other imaging modalities or anatomical atlases.

As Microscopy-BIDS supports the OME-TIFF file format, which includes metadata in its header, it was understood that some metadata would be redundant between the OME-TIFF and the sidecar JSON metadata. Our criteria for metadata were to (i) incorporate the most common metadata fields necessary for image analysis and (ii) avoid unnecessary metadata duplication when possible, as duplicates would need to be validated for consistency and could cause errors or delays in the curation process. Moreover, a complete list of metadata covering every modality and use case could be rather burdensome and overwhelming. To promote usability and adoption of the standard, and given that the microscopy community has not reached a consensus on metadata reporting, we chose to include only the most common and established metadata fields requested by our contributors, but made sure our proposal was compatible with other initiatives. For examples, the Microscopy-BIDS definition of a “sample” is compatible with the SPARC data structure (Bandrowski et al., 2021), and the majority of the required “Specimen,” “Image,” and “Instrument” metadata from the Essential Metadata for 3D BRAIN Microscopy (Ropelewski et al., 2021)^28^ can find a place in Microscopy-BIDS, except for fields that may overlap with future coordinate system descriptions. Additionally, any other subject or sample attributes can be described in the “participants.tsv” and “samples.tsv” metadata files. For image acquisition, we included an additional “OtherAcquisitionParameters” field allowing the description of other relevant image acquisition parameters for particular cases. Similarly, both the image acquisition and sample JSON metadata sections include a field allowing the description of protocols that could embed additional information.

Like many standards, Microscopy-BIDS is not frozen in time. The items/specifications listed in this article correspond to BIDS version 1.7.0 and are likely to evolve with new microscopy modalities and standardization initiatives. Those future developments could include additional metadata reporting for quality assessment and processing pipelines, which are not covered by the current specification focusing on raw data. As microscopy data formats are constantly evolving, other file formats may be supported in the future. For example, the OME consortium has recently developed the Next-Generation File Formats (OME-NGFF) as a successor to OME-TIFF for better remote sharing of large datasets in cloud-based resources (Moore et al., 2021). Support for this format has been discussed and will be incorporated after the next version is released, which includes substantial changes related to incorporating additional metadata about describing the axes and related spatial coordinate systems. Future works also include the development of tools for microscopy data conversion to BIDS format and the standardization for microscopy derivatives.

The Microscopy-BIDS specification is complemented by an extension to the *bids-validator* and example datasets. Its standard file naming scheme and data structure, including human- and machine-readable metadata, will facilitate the sharing of microscopy data and multi-modal data analysis with other BIDS modalities.

## Genesis of the Microscopy Extension Proposal

An initial microscopy data structure was proposed in the Neurostars forum^29^ and BIDS mailing list^30^ in June 2020. Following feedback from the community, a first version was drafted in the form of a Google document following the BIDS guidelines,^31^ which then became the official BIDS extension proposal (BEP031) in August 2020. Feedback from the community (40+ researchers in the field) was requested and integrated in the proposal throughout the end of 2020, aiming for consensus. A series of virtual meetings took place during the winter and spring of 2021 to discuss and finalize the finer details of the proposal. In parallel, discussions were held in collaboration with the animal electrophysiology extension proposal (BEP032) and the BIDS community to incorporate animal metadata to the specification as well as describe multiple samples from the same subject. The microscopy BEP031 underwent community review on GitHub in November 2021 and was incorporated into the BIDS-specification as part of the release 1.7.0 in February 2022.

## Data Availability Statement

The links to the example data presented in the study are included in the article, further inquiries can be directed to the corresponding author.

## Members of the BIDS Maintainers

Stefan Appelhoff, Ross Blair, Eric Earl, Franklin Feingold, Anthony Galassi, Rémi Gau, Christopher J. Markiewicz, and Taylor Salo.

## Author Contributions

M-HB and JC-A wrote the original manuscript, made the figures, developed the initial draft of the proposal, and managed contributions from the community. M-HB and the BIDS Maintainers merged the extension proposal to the main BIDS specification. JC-A supervised the project. M-HB, LK, SG, GM, AL, CM, RO, YH, IL, ST, P-JT, AK, FC, the BIDS Maintainers, and JC-A contributed to the meetings. M-HB, LK, SG, GM, AL, CM, RO, GNis, YH, IL, ST, P-JT, AK, GNil, the BIDS Maintainers, and JC-A contributed to the writing and/or critical review of the proposal. M-HB, GM, FC, and JC-A contributed datasets. M-HB contributed to preparing example datasets and developing the validator. All authors contributed to the manuscript and approved the submitted version.

## Author Disclaimer

The content is solely the responsibility of the authors and does not necessarily represent the official views of the National Institutes of Health.

## Conflict of Interest

The authors declare that the research was conducted in the absence of any commercial or financial relationships that could be construed as a potential conflict of interest.

## Publisher’s Note

All claims expressed in this article are solely those of the authors and do not necessarily represent those of their affiliated organizations, or those of the publisher, the editors and the reviewers. Any product that may be evaluated in this article, or claim that may be made by its manufacturer, is not guaranteed or endorsed by the publisher.

## Footnotes

1
https://braininitiative.nih.gov

2
https://github.com/HumanBrainProject/openMINDS

3
https://docs.openmicroscopy.org/ome-model/6.2.2

4
https://www.4dnucleome.org

5
https://www.bioimagingna.org

6
https://bids.neuroimaging.io

7
http://bids-apps.neuroimaging.io

8
https://bids.neuroimaging.io/benefits.html#software

9
https://github.com/bids-standard/bids-validator

10
https://bids-specification.readthedocs.io/en/stable/04-modality-specific-files/10-microscopy.html

11
https://docs.openmicroscopy.org/ome-model/6.2.2/ome-tiff/specification.html

12
https://docs.openmicroscopy.org/ome-model/6.2.2/ome-tiff/data.html

13
https://docs.openmicroscopy.org/ome-model/6.2.2

14
https://www.openmicroscopy.org/bio-formats

15
https://github.com/cgohlke/tifffile

16
https://github.com/libvips/libvips

17
https://github.com/apeer-micro/apeer-ometiff-library

18
https://github.com/filippocastelli/pyometiff

19
https://bids-specification.readthedocs.io/en/stable/02-common-principles.html#definitions

20
https://www.encodeproject.org/profiles/biosample_type

21
https://www.ncbi.nlm.nih.gov/Taxonomy/Browser/wwwtax.cgi

22
https://scicrunch.org/resources

23
https://dicom.nema.org/medical/dicom/current/output/chtml/part16/chapter_L.html#chapter_L

24
https://github.com/bids-standard/bids-validator

25
https://github.com/bids-standard/bids-examples#microscopy-datasets

26
https://dandiarchive.org/dandiset/000026

27
https://dandiarchive.org/dandiset/000108

28
https://doryworkspace.org/metadata

29
https://neurostars.org

30
https://groups.google.com/g/bids-discussion

31
https://bids.neuroimaging.io/get_involved.html
